# Small bowel lymphoma on small bowel endoscopic ultrasound

**DOI:** 10.1093/gastro/goae054

**Published:** 2024-06-17

**Authors:** Zhongcheng Liu, Zicheng Huang, Bo Peng, Ting Xiao, Miao Li, Qin Guo

**Affiliations:** Department of Small Bowel Endoscopy, the Sixth Affiliated Hospital, Sun Yat-sen University, Guangzhou, Guangdong, P. R. China; Biomedical Innovation Center, the Sixth Affiliated Hospital, Sun Yat-sen University, Guangzhou, Guangdong, P. R. China; Biomedical Innovation Center, the Sixth Affiliated Hospital, Sun Yat-sen University, Guangzhou, Guangdong, P. R. China; Department of Gastroenterology, the Sixth Affiliated Hospital, Sun Yat-sen University, Guangzhou, Guangdong, P. R. China; Guangdong Provincial Key Laboratory of Colorectal and Pelvic Floor Diseases, the Sixth Affiliated Hospital, Sun Yat-sen University, Guangzhou, Guangdong, P. R. China; Department of Small Bowel Endoscopy, the Sixth Affiliated Hospital, Sun Yat-sen University, Guangzhou, Guangdong, P. R. China; Biomedical Innovation Center, the Sixth Affiliated Hospital, Sun Yat-sen University, Guangzhou, Guangdong, P. R. China; Department of Small Bowel Endoscopy, the Sixth Affiliated Hospital, Sun Yat-sen University, Guangzhou, Guangdong, P. R. China; Biomedical Innovation Center, the Sixth Affiliated Hospital, Sun Yat-sen University, Guangzhou, Guangdong, P. R. China; Biomedical Innovation Center, the Sixth Affiliated Hospital, Sun Yat-sen University, Guangzhou, Guangdong, P. R. China; Department of Gastroenterology, the Sixth Affiliated Hospital, Sun Yat-sen University, Guangzhou, Guangdong, P. R. China; Guangdong Provincial Key Laboratory of Colorectal and Pelvic Floor Diseases, the Sixth Affiliated Hospital, Sun Yat-sen University, Guangzhou, Guangdong, P. R. China; Department of Small Bowel Endoscopy, the Sixth Affiliated Hospital, Sun Yat-sen University, Guangzhou, Guangdong, P. R. China; Biomedical Innovation Center, the Sixth Affiliated Hospital, Sun Yat-sen University, Guangzhou, Guangdong, P. R. China; Department of Gastroenterology, the Sixth Affiliated Hospital, Sun Yat-sen University, Guangzhou, Guangdong, P. R. China; Guangdong Provincial Key Laboratory of Colorectal and Pelvic Floor Diseases, the Sixth Affiliated Hospital, Sun Yat-sen University, Guangzhou, Guangdong, P. R. China

## Introduction

There are only a few cases of small bowel lymphoma diagnosed by small bowel endoscopic ultrasonography (EUS) described in the literature. Here, the clinical features, enteroscopic manifestations, small bowel endoscopic ultrasound manifestations, and treatment of small bowel lymphoma are described.

## Patients and methods

This study retrospectively analysed patients with small bowel lymphoma admitted between April 2023 and October 2023. All patients underwent a double balloon enteroscopy (DBE; EN-580T, Fujifilm, Japan) and small bowel EUS (Endoscopic Ultrasound System IM-02P-202501, InnerMedical Co., Ltd, Shenzhen, China) (ethics number: 2023ZSLYEC-264).

## Results

### Case 1

**Figure 1.**
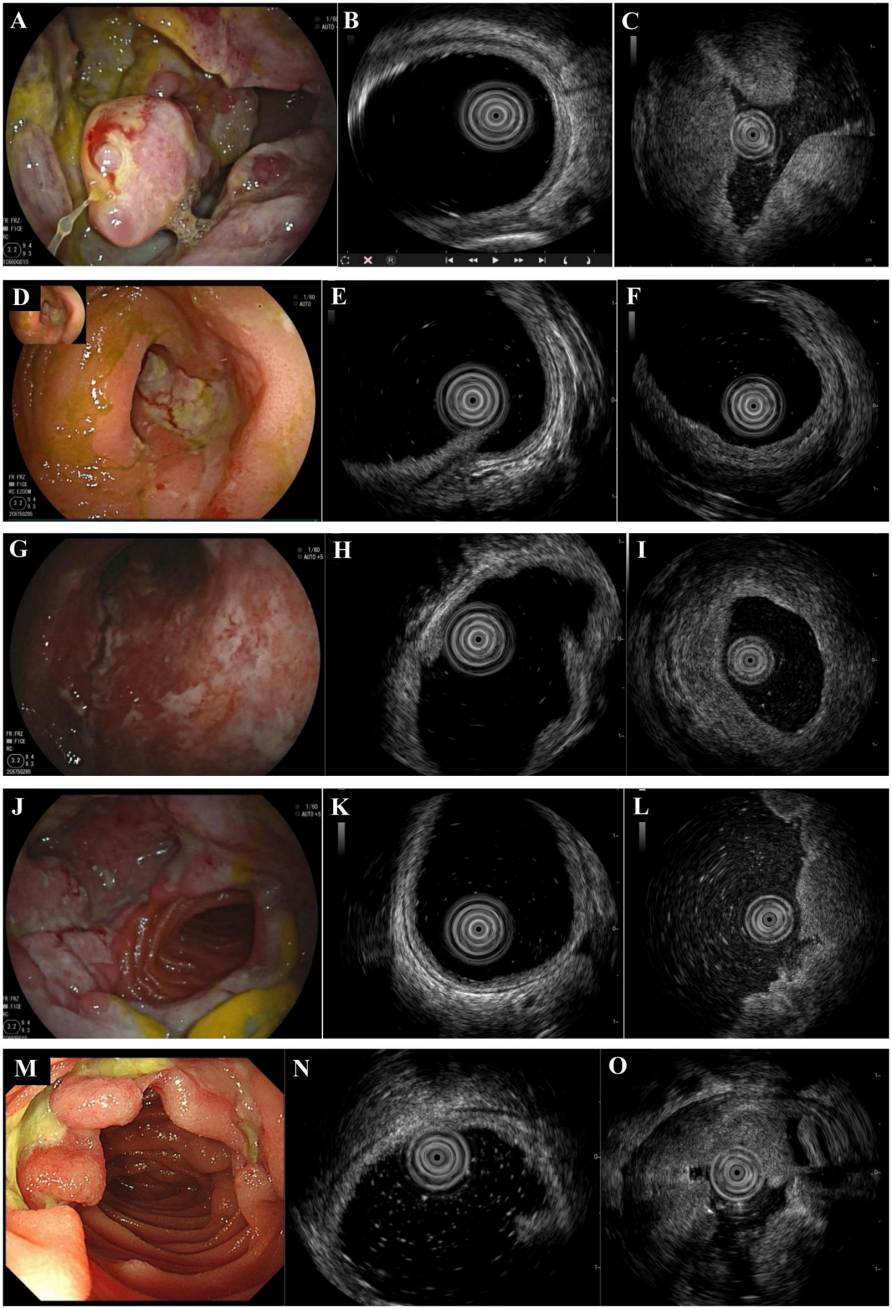
Clinical data of five cases included in this study. (**A**) DBE shows a bulging, irregular mass ∼10 cm long in the distal jejunum; the surface of the mass is covered with white mucus (Case 1). (**B**) Normal small intestinal wall on EUS shows no thickening of the intestinal wall (Case 1). (**C**) EUS shows thickening of the entire bowel wall, fusion and loss of demarcation of bowel wall layers, hypoechoic changes in some areas, and even lower echogenicity inside the hypoechoic areas (Case 1). (**D**) DBE shows a longitudinal lamellar ulcer with a thick white coating and congestion of the mucosa surrounding the ulcer in the proximal ileum (Case 2). (**E**) Normal small intestinal wall on EUS shows no thickening of the intestinal wall (Case 2). (**F**) EUS shows thickening of the entire intestinal wall, indistinct demarcation between the mucosal and submucosal layers in some areas, and localized hypoechoic or hyperechoic changes in the submucosa and muscularis propria (Case 2). (**G**) DBE showed a bulging, circumferential mass ∼15 cm long with a white coating and congestion in the proximal ileum (Case 3). (**H**) Normal small intestinal wall on EUS shows no thickening of the intestinal wall (Case 3). (**I**) EUS shows thickening of the entire bowel wall, fusion of bowel wall layers, indistinct demarcation between the submucosa and muscularis propria, hypoechoic changes in some areas of the submucosa, and even lower echogenicity inside the hypoechoic areas within muscularis propria (Case 3). (**J**) DBE shows an irregular, elevated, circumferential mass ∼10 cm long with a yellowish-white coating (Case 4). (**K**) Normal small intestinal wall on EUS shows no thickening of the intestinal wall (Case 4). (**L**) EUS shows that the entire bowel wall is thickened, and there is fusion and loss of demarcation of bowel wall layers (Case 4). (**M**) DBE shows multiple irregular, depressed, debris-covered ulcers starting from the distal duodenum to the proximal jejunum (Case 5). (**N**) Normal small intestinal wall on EUS shows no thickening of the intestinal wall (Case 5). (**O**) EUS shows that the entire bowel wall is thickened, predominantly in the muscularis propria (Case 5). DBE = double balloon enteroscopy, EUS = endoscopic ultrasonography.

A 72-year-old male was admitted because of intermittent black stools for over a year. The manifestations of DBE and small bowel EUS are shown in Figure 1A–C. Pathology showed lymphoid cells were diffusely distributed in the mucosal tissues; the cytosol was predominantly small- to medium-sized; large cells with round or irregular nuclei were observed. The patient received surgical operation. Postoperative pathology findings indicated that diffuse proliferation of lymphoid cells in the entire bowel wall was seen in the whole layer of the intestinal wall. The cells were monomorphic and medium-sized, with an intermediate amount of cytoplasm and round or oval nuclei, accompanied by necrosis. Epitheliotropic manifestations were observed in some sites. The lesion was diagnosed as a highly invasive T-cell lymphoma consistent with monomorphic epitheliotropic intestinal T-cell lymphoma. No tumour involvement was observed in the resection margins.

### Case 2

A 49-year-old male was admitted because of intermittent black stools for half a year. The manifestations of DBE and small bowel EUS are shown in Figure 1D–F. The patient received surgical operation. Pathology showed that many lymphoid cells with a diffuse or nodular distribution were observed in all layers of the intestinal wall, suggesting the possibility of a lymphohematopoietic tumour. Thus, the patient was diagnosed as having mucosa-associated tissue lymphoma.

### Case 3

A 68-year-old female was admitted because of recurrent abdominal pain for over a year. The manifestations of DBE and small bowel EUS are shown in Figure 1G–I. Pathology showed that infiltration of heterogeneous lymphoid cells with occasional mitotic figures was observed; the patient was suggestive of an anaplastic variant of diffuse large B cell lymphoma. The patient received rituximab + cyclophosphamide + doxorubicin + vincristine + prednisone regimen.

### Case 4

A 48-year-old male was admitted because of black stools for 6 months. The manifestations of DBE and small bowel EUS are shown in Figure 1J–L. Pathology showed that, in the small bowel mucosa tissue, large, markedly heterogeneous lymphoid cells were observed to be diffusely distributed in sheets. The patient received a surgical operation.

### Case 5

A 51-year-old male was admitted because of black stools for a year. The manifestations of DBE and small bowel EUS are shown in Figure 1M–O. Pathology showed the small bowel mucosa tissue had atrophied crypts, blunted villi, and highly heterogeneous lymphoid cells in the interstitium; some nucleoli and apoptosis were observed. Peripheral T-cell lymphoma was considered highly probable. The patient received vincristine + cyclophosphamide + epirubicin + dexamethasone + etoposide + tucidinostat regimen.

## Discussions

Primary small intestinal lymphomas are malignant tumours originating from the lymphoid tissue of the intestinal lamina propria and submucosa, and they account for 19%–38% of small bowel malignancies and 20%–30% of all primary gastrointestinal lymphomas [1]. Early symptoms of primary small intestinal lymphoma are insidious and lack specificity, and the treatment plan and prognosis are directly dependent on the accuracy of clinical staging and whether it is diagnosed at an early stage. Thus, a preoperative diagnosis is essential.

Small bowel EUS is performed through the lumen of the intestinal tract. The ultrasound probe directly contacts the lesion, minimizing their distance from each other, thereby preventing interference from intestinal gas, reducing echo signal attenuation, and increasing resolution, thus yielding the histological characteristics of the various layers of the bowel and producing endoscopic ultrasonograms of the surrounding organs [2].

Taking together the experience at our centre, we classify the small bowel endoscopic ultrasound presentation of small bowel lymphoma into two types on the basis of enteroscopic presentation: (i) limited or diffuse circumferential thickening of the bowel wall with homogeneous hypoechogenicity and loss of its normal layered organization, as observed in Cases 1, 3, and 4; and (ii) thickening of the entire bowel wall but without fusion, presenting as thickening of the muscularis propria, as observed in Cases 2 and 5. The differential diagnosis includes other small bowel diseases such as small bowel mesenchymal tumours, adenocarcinoma, Crohn’s disease, and gastrointestinal tuberculosis.

We advanced our centre’s experience and partially summarized the small bowel endoscopic ultrasound presentation of small bowel disease as follows. Small bowel mesenchymal tumours are often masses that grow towards the lumen and outward and are large in volume. Larger tumours are often accompanied by haemorrhage, necrosis, or cystic degeneration. On small bowel EUS, small bowel disease presents as hypoechoic changes with clear borders, often appearing round and originating from the muscularis propria in most cases. However, they can also originate from the mucosa. Small bowel adenocarcinoma lesions are often more limited, tending to develop in the proximal small bowel with local stiffness of the bowel wall and narrowing of the lumen, and they are often accompanied by enlarged lymph nodes surrounding the lesion. In the presence of invasive blood vessels, the strong echogenicity of the fat surrounding the blood vessels disappears. When the blood vessels are partially or completely encapsulated by the mass, necrosis of the bowel wall layers is observed on small bowel EUS as heterogeneous hypoechogenicity and rupture of the serosa. The small bowel endoscopic ultrasound presentation of Crohn’s disease involves different degrees of thickening in different layers of the bowel wall, predominantly in the submucosa and the muscularis propria.

Additionally, the submucosa may exhibit slight hyperechoic or hypoechoic changes. Layers can be distinguished, but the demarcation is poor. The structure of the submucosa and the muscularis propria is lost at the ulcer site. Intestinal tuberculosis presents on small bowel EUS as the absence of the mucosa at the ulcer site, thickening of the bowel wall surrounding the lesion, predominantly the muscularis propria, and thinning of the submucosa with decreased echogenicity.

There are few cases of small bowel lymphoma diagnosed by small bowel EUS described in the literature, but we expect future data to confirm the present study’s results.

## Authors’ contributions

Z.L. was responsible for study design, data collection, data analysis, writing of the manuscript and the integrity of the work from inception to published article. Z.H., B.P., T.X., and M.L. were responsible for study design, data collection, and data analysis. Q.G. was responsible for patient recruitment and data collection. All authors read and approved the final version of the manuscript.
